# Transgenic overexpression of *GmAPC7-CT* improves seed yield and reduces susceptibility to soybean mosaic virus and *Meloidogyne incognita* in soybean

**DOI:** 10.1007/s00425-026-05097-6

**Published:** 2026-09-07

**Authors:** Marcos Fernando Basso, Stéfanie Menezes de Moura, Daniele Heloísa Pinheiro, Thuanne Pires Ribeiro, Nayara Sabrina Freitas-Alves, Karina Nascimento Silva Fragoso, Rodrigo Rocha Fragoso, Bruna Gino de Araújo-Lopes, Adriana Silva Hemerly, Janice de Almeida-Engler, Maria Fatima Grossi-de-Sa

**Affiliations:** 1 Embrapa Genetic Resources and Biotechnology, PqEB Final, W5 Norte, PO Box 02372, Brasília, DF 70770-917 Brazil; 2 National Institute of Science and Technology, INCT PlantStress Biotech, EMBRAPA, Brasília, DF Brazil; 3 Federal University of Rio de Janeiro, Rio de Janeiro, RJ Brazil; 4 INRAE, Université Côte d’Azur, CNRS, Sophia-Antipolis, ISA France; 5 Catholic University of Brasília, Brasília, DF Brazil; 6 Dom Bosco Catholic University, Campo Grande, MS Brazil

**Keywords:** Anaphase-promoting complex, *Glycine max*, Glyma.15G096000, Plant biotechnology, Root-knot nematode, Soybean mosaic disease, *Potyvirus*

## Abstract

**Main conclusion:**

Stable transgenic soybean lines overexpressing the *GmAPC7-CT* gene have demonstrated increased seed yield and reduced susceptibility to the soybean mosaic virus and *Meloidogyne incognita*.

**Abstract:**

The Anaphase-Promoting Complex subunit 7 (APC7) is a core structural component of the anaphase-promoting complex or cyclosome (APC/C). The terminal region of this *AtAPC7* gene has been shown in *Arabidopsis thaliana* to accumulate more transcripts than the full-length gene. The *AtAPC7-CT* gene (terminal region of the *AtAPC7*) encodes a protein with significant homology to a tobacco viral replication inhibitor (IVR). Its stable overexpression in transgenic *A. thaliana* lines resulted in notable improvements in biomass, seed yield, earliness of vegetative-reproductive transitions, and reduced susceptibility to viruses. In this study, we generated stable transgenic soybean lines overexpressing the *GmAPC7-CT* gene (terminal region or 3’ portion of Glyma.15G096000, corresponding to the *AtAPC7-CT*) and evaluated seed yield and susceptibility of these lines to soybean mosaic virus and *Meloidogyne incognita*. The *GmAPC7-CT* gene is 624 nucleotides long and encodes a 207-amino acid protein with two tetratricopeptide repeat (TPR) domains. GmAPC7-CT showed 100% amino acid identity with full-length GmAPC7, 81.16% identity with AtAPC7-CT, and 87.94% identity with tobacco IVR. Stable transgenic lines demonstrated significant advancements in plant development and seed yield, with the top three lines producing up to 43% more pods, 44% more seeds, and a 16% increase in seed weight. Furthermore, these soybean lines showed up to a 70% reduction in susceptibility to soybean mosaic virus and *M. incognita*, reflected by decreased viral RNA load and nematode reproduction factor. Collectively, these results support a conserved role of GmAPC7-CT in soybean and AtAPC7-CT in *A. thaliana*, acting similarly to the tobacco IVR. Thus, our findings underscore the strong biotechnological potential of the *GmAPC7-CT* gene to improve key agronomic traits in soybean through genetic engineering approaches, including conventional breeding, transgenesis, and genome editing.

**Supplementary Information:**

The online version contains supplementary material available at https://doi.org/10.1007/s00425-026-05097-6.

## Introduction

Soybean (*Glycine max*) is one of the most important agricultural commodities in the world and serves as a vital raw material for human and animal nutrition (Hartman et al. 2011). Brazil, the USA, and Argentina are the largest soybean producers, with Brazil being the leading exporter and China the primary importer (Basso et al. 2024; Grossi-de-Sa and Basso 2024; Tetrault 2024). Soybean is susceptible to numerous abiotic stresses, pathogens, and insect pests that cause significant yield losses (Nakayama et al. 2017; Savary et al. 2019; Roth et al. 2020). In particular, plant-parasitic nematodes and viruses are significant constraints on soybean production (Mazzetti et al. 2019; Roth et al. 2020; Horikoshi et al. 2021). Soybean mosaic virus (genus *Potyvirus*, family Potyviridae) has soybean as its main host and is transmitted in a non-persistent manner by aphids and through infected seeds (Hajimorad et al. 2018). Foliar symptoms range from asymptomatic or moderate to severe, with leaf mottling, leaf distortion, necrosis, and stunting in symptomatic susceptible cultivars (Liu et al. 2016; Maugeri et al. 2025). Yield losses depend on the soybean genotype or cultivar, virus strain or isolate, infection incidence, and soybean development stage, but losses of up to 94% have already been reported in uniformly infected field plots (Hill and Whitham 2014). Root-knot nematodes (RKNs), such as *Meloidogyne incognita* (genus *Meloidogyne*), are among the most economically important species infecting soybean and causing significant yield losses (Mazzetti et al. 2019). Symptoms in soybean include gall-like formations on the roots, which damage the root system and can cause losses of up to 90% in cultivated areas with a high incidence (Jones et al. 2013). The *M. incognita* life cycle includes the following stages: egg, ppJ2 (pre-parasitic second-stage juveniles), pJ2 (parasitic second-stage juveniles), J3 and J4 non-feeding juveniles, and adult females (Castagnone-Sereno et al. 2013). Therefore, the development of new biotechnological tools based on conventional breeding, transgenesis, or genome editing to improve soybean yield and reduce plant susceptibility to these pathogens is an urgent need for farmers (Basso et al. 2019, 2020; Freitas-Alves et al. 2024).

The Anaphase-Promoting Complex or Cyclosome (APC/C) is one of the main cellular mechanisms that orchestrates the cell cycle in plants (McLean et al. 2011; Eloy et al. 2015; Gonçalves et al. 2026). These successive events act in a highly ordered manner during the progression of the cell cycle (Saleme et al. 2021; Gonçalves et al. 2026). The APC/C catalytic core is composed of several APC subunits (Tang et al. 2001; Fülöp et al. 2005; Fonseca et al. 2011). The APC7 subunit contains tetratricopeptide repeat (TPR) domains, which are important for protein–protein interaction and coupling in the APC/C (Eloy et al. 2006; Van Leene et al. 2010; Carneiro et al. 2021; Wang et al. 2026). APC/C subunits have been described as closely associated with plant growth and development (Rojas et al. 2009; Lima et al. 2010, 2013; Eloy et al. 2011; Schwedersky et al. 2021; Oliveira et al. 2022; Wang et al. 2026). The role of the full-length AtAPC7 protein in the cell cycle has recently been discussed, highlighting that the functional implications of the higher expression of the *AtAPC7-CT* gene, compared to the full-length *AtAPC7* gene, are associated with plant growth (Eloy et al. 2006; Araújo-Lopes et al. 2025). The overexpression of the full-length *AtAPC7* gene in transgenic *A. thaliana* correlated with plants exhibiting a greater number of cells, as well as increased numbers and sizes of epidermal and parenchymal cells, leaf area, endoreduplication index in leaves, growth, development, root system, shoot branching, chlorophyll and carotenoid contents, stomatal index, photosynthetic efficiency, biomass yield, earliness in vegetative-reproductive transition, and seed yield (Araújo-Lopes et al. 2025). Furthermore, the APC7-CT protein has been implicated in plant growth and development and in inhibiting DNA and RNA virus replication (Araújo-Lopes et al. 2025). Similarly, transgenic overexpression of the *NtAPC7-CT* gene in *Nicotiana tabacum* resulted in increased plant resistance to tobacco mosaic virus and *Botrytis cinerea* (Akad et al. 2005). These promising results from the overexpression of the *AtAPC7-CT* and *NtAPC7-CT* genes in model plants justify further investigation to determine whether these findings can be applied to major crops, such as soybean.

In this study, the *GmAPC7-CT* gene was identified in the soybean genome, and stable transgenic soybean events overexpressing this gene were generated via genetic transformation mediated by *Agrobacterium tumefaciens*. Ten independent transgenic lines were obtained and phenotyped for plant development and seed yield under greenhouse conditions. The three top-performing transgenic lines, selected based on superior seed yield, were subsequently challenged with soybean mosaic virus and *M. incognita*. The combined increase in seed yield and reduction in susceptibility to these two major pathogens are discussed, emphasizing the strategic biotechnological value of the *GmAPC7-CT* as a dual-function gene to simultaneously enhance productivity and resilience in soybean.

## Materials and methods

### *GmAPC7-CT* gene sequence and binary vector

**Fig. 1:**
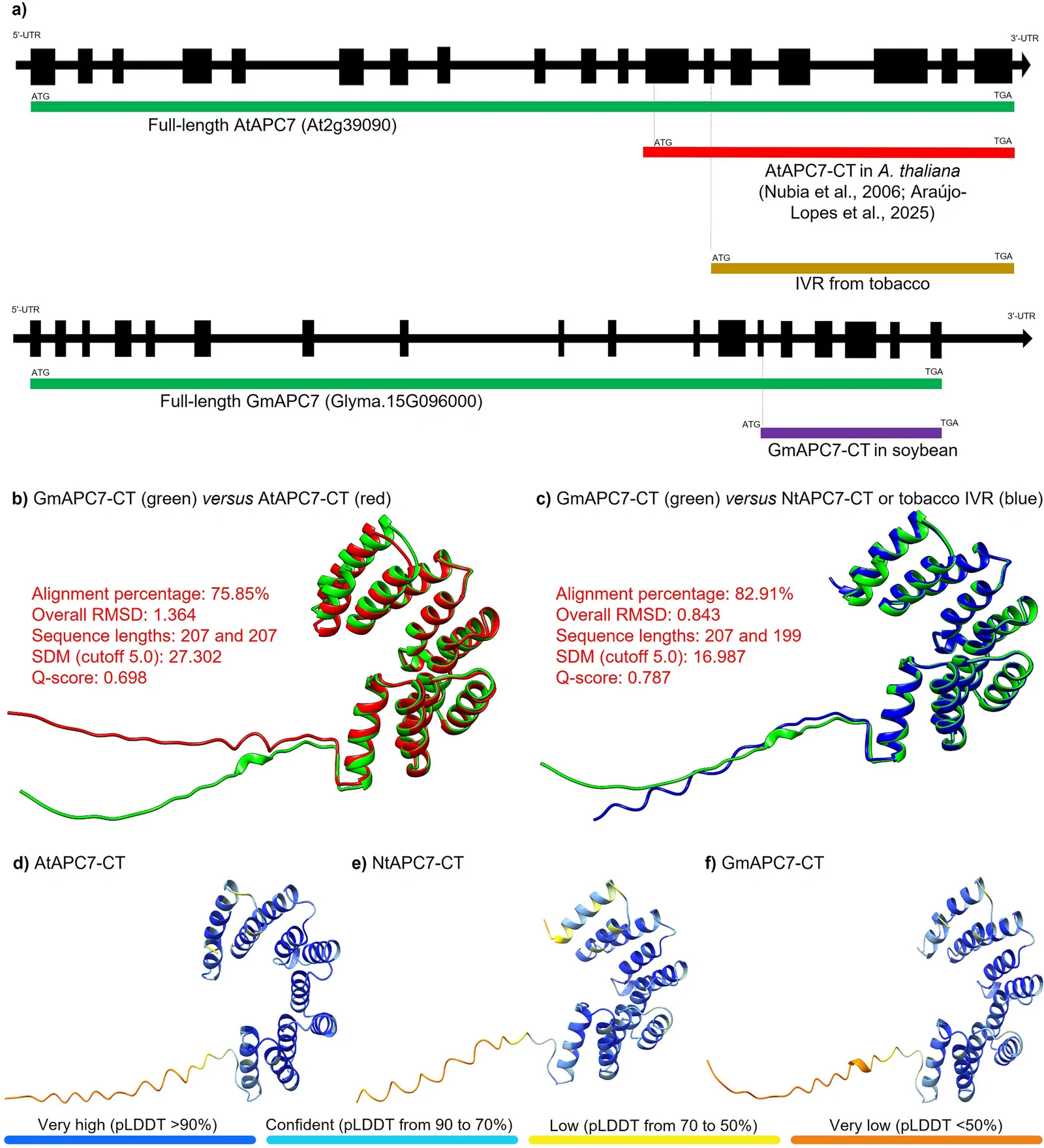
*GmAPC7-CT* engineering from the full-length *GmAPC7* gene and based on *AtAPC7-CT* and viral replication inhibitor (IVR) of tobacco. **a** The GmAPC7-CT was determined based on sequence alignment with AtAPC7-CT and tobacco IVR. **b**, **c** Comparison of the tertiary structure of the GmAPC7-CT *versus* AtAPC7-CT (**b**) and GmAPC7-CT *versus* NtAPC7-CT or tobacco IVR proteins provided by AlphaFold v.3 (**c**) **d**-**f** Three-dimensional structure of the AtAPC7-CT (**d**), NtAPC7-CT (**e**), and GmAPC7-CT (**f**) proteins. Regions of different confidence degrees are expressed with different colors according to the predicted local distance difference test (pLDDT), ranging from dark blue (very high confidence degree, pLDDT > 90%), cyan (confident degree, pLDDT from 90 to 70%), yellow (low confidence degree, pLDDT from 70 to 50%), to red (very low confidence degree, pLDDT < 50%)

The full-length *GmAPC7* (Glyma.15G096000) and *GmAPC7-CT* genes were retrieved from the Phytozome v.13 database (Goodstein et al. 2012) using the *AtAPC7-CT* gene (At2g39090) sequence as a reference (Suppl. File S1). The BLASTn and BLASTp tools (NCBI) were used for sequence homology analysis (Altschul et al. 1990). The tertiary structures of the GmAPC7-CT *versus* AtAPC7-CT and NtAPC7-CT proteins were predicted and compared by AlphaFold v.3 (Abramson et al. 2024). The predicted subcellular localization and the predicted membrane association were performed using the DeepLoc 2.1 software (Thumuluri et al. 2022). The *GmAPC7-CT* sequence was synthesized and assembled into a binary vector backbone by Epoch Life Science (Sugar Land, TX, USA). The T-DNA was composed of the *GmAPC7-CT* gene controlled by the soybean pUceS8.3 promoter (Fragoso et al. 2022) and TNOS terminator, the synthetic *acetohydroxyacid synthase* (*ahas*) was used as a selectable marker gene (Rech et al. 2008) controlled by the AHAS promoter and AHAS terminator of *A. thaliana* (Vianna et al. 2011). The *PAT/bar* (phosphinothricin acetyltransferase/bialaphos) was used as a second selectable marker gene controlled by the CaMV 35S promoter and TNOS terminator for plant screening under greenhouse conditions (Fig. 1b). The pUceS8.3::GmAPC7-CT binary vector was transfected into *Escherichia coli* strain DH5α and, subsequently, transferred to *A. tumefaciens* strain GV3101.

### Genetic transformation of soybean and characterization of transgenic plants

Embryonic axes were isolated from the soybean cv. BR143465 and used for genetic transformation combining biolistic and *Agrobacterium*-mediated methods, as described by Paes-de-Melo et al. (2020).In vitro plant regeneration was conducted in a selective medium containing 600 nM of Imazapyr herbicide (Sigma-Aldrich, Saint Louis, MO, USA). The recovered and rooted plants were acclimatized and maintained under greenhouse conditions. Transgenic soybean plants were screened by PCR for the presence of *PAT/bar* and *GmAPC7-CT* genes using *PAT/bar*-specific primers (490 bp amplicon) and pUceS8.3::GmAPC7-CT-specific primers (457 bp amplicon) (Suppl. Table S1). Genomic DNA was extracted from young leaves using the DNeasy Plant Maxi kit (Qiagen, Hilden, Germany) according to the manufacturer’s instructions. Its concentration was determined using a NanoDrop 2000 spectrophotometer (Thermo Fisher Scientific, Waltham, MA, USA), and its integrity was confirmed by 0.8% (w/v) agarose gel electrophoresis. PCR assays were conducted using the Extract-N-Amp Plant PCR Kit (Qiagen), 20 ng of genomic DNA, and 10 mM target-specific primers (Suppl. Table S1). Genomic DNA purified from non-transgenic plants served as a negative control, while the pUceS8.3::GmAPC7-CT binary vector served as a positive control in the PCR. Furthermore, the PAT/bar protein was also detected in transgenic soybean plants by indirect ELISA (Enzyme-Linked Immunosorbent Assay) using an HRP-conjugated anti-PAT/bar polyclonal antibody (MBS1491343; MyBioSource, San Diego, CA, USA). Seeds were collected individually from each transgenic event, and generations were advanced from T_0_ to T_4_ and screened by PCR and indirect ELISA assays. Protein extraction, quantification, and indirect ELISA procedures were performed as described by Ribeiro et al. (2020, 2024). Ten transgenic soybean lines from the T_4_ generation were obtained and phenotyped for plant growth, earliness, and seed yield under greenhouse conditions. From these, three transgenic soybean lines with the best performance, based on seed yield, were selected for subsequent evaluation of plant susceptibility to soybean mosaic virus and *M. incognita*.

### *GmAPC7-CT* gene expression in transgenic soybean lines

Soybean leaf samples from T_2_ transgenic lines were ground in liquid nitrogen with a mortar and pestle, and total RNA purification was performed using Concert Plant RNA Reagent (Invitrogen, Carlsbad, CA, USA) supplemented with PVP-40. Total RNA samples were treated with RNase-free RQ1 DNase I (Promega, Madison, WI, USA). Subsequently, 2 μg DNase-treated RNA was used as a template for cDNA synthesis with oligo (dT)_20_ primer and SuperScript III RT kit (Invitrogen). The real-time PCR assays were performed in a QuantStudio 3 Real-Time PCR system (Applied Biosystems, Waltham, MA, USA) using 200 ng of cDNA, 0.2 µM of each gene-specific primer (Suppl. Table S1), and GoTaq qPCR Master Mix (Promega). The thermocycling program used was 50ºC for 2 min, 95ºC for 10 min, 40 cycles of 95ºC for 15 s and 60ºC for 1 min, and melting curve analysis using the standard program. All cDNA samples were prepared in technical triplicate reactions. Target-specific amplifications were confirmed by a single, distinct peak in the melting curve analysis. The relative expression was calculated using the formula 2^−∆Ct^ (Rao et al. 2013), and *GmELF1A* was used as an endogenous reference gene for expression normalization (Suppl. Table S1; Miranda et al. 2013). *GmCYP2* was also tested as an endogenous reference gene, but *GmELF1A* was more stable in these samples.

### Assessing phenotypic traits in transgenic soybean lines

Seeds of transgenic soybean lines from the T_4_ generation and non-transgenic soybean plants were germinated on Germitest paper, and 20–25 uniform plants were transplanted into bags containing 5 kg soil and substrate mixture (2:1; w:w) and maintained under greenhouse conditions at 25 ± 10°C and 50% humidity. Plants were irrigated regularly to maintain soil moisture near field capacity and fertilized according to standard soybean cultivation practices with macro- and micronutrients. Plant developmental parameters were evaluated, including vegetative biomass, root biomass, number of pods per plant, number of seeds per plant, and seed weight per plant. The parameters were evaluated in at least 20 plants from each soybean transgenic line compared with non-transgenic plants.

### Susceptibility level of transgenic soybean lines to soybean mosaic virus

At least 21 plants per transgenic line from the T_4_ generation were used for each treatment, while 21 non-transgenic soybean plants cv. BR143465 were used as a negative control. Two sets of non-transgenic plants were mock- or virus-inoculated. All plants were maintained in bags containing soil and substrate mixture (2:1; w:w) under greenhouse conditions at 25 ± 10°C and 50% humidity. Plants were irrigated regularly and fertilized according to standard soybean cultivation practices as described above. Young transgenic and non-transgenic soybean plants were mechanically inoculated with the leaf extract of soybean mosaic virus-infected soybean plants using carborundum as an abrasive and inoculation buffer (50 mM K_2_HPO_4_ and 0.1% Na_2_SO_3_, pH 7.5). All plants were re-inoculated after three days to reduce the likelihood of infection failure resulting from inoculation. Furthermore, soybean plants of the genotype PI595099 (symptomatic susceptible) and cultivar BRS133 (asymptomatic susceptible) were used as controls for viral inoculation (Suppl. Fig. S1). At 20 days after inoculation (DAI), leaf samples were collected, comprising three biological repetitions, each representing seven plants, kept in liquid nitrogen and macerated with a pestle and mortar. Total RNA was purified using Concert Plant RNA Reagent (Invitrogen) supplemented with PVP-40. RNA samples were treated with RNase-free RQ1 DNase I (Promega) and, subsequently, 1.5 μg DNase-treated RNA was used as a template for cDNA synthesis as described above. Real-time PCR assays were performed with a QuantStudio 3 Real-Time PCR System (Applied Biosystems) using 250 ng of cDNA, 0.15 µM of each gene-specific primer (Suppl. Table S1), and GoTaq qPCR Master Mix (Promega). The thermocycling process used is the same as described above. All cDNA samples were performed in technical triplicate reactions. Target-specific amplifications were confirmed by a single, distinct peak in the melting curve analysis. Relative accumulation of viral RNA was calculated using the formula 2^−∆Ct^ (Rao et al. 2013), while the *GmCYP2* was used as an endogenous reference gene for viral load normalization (Suppl. Table S1; Miranda et al. 2013). *GmELF1A* was also tested as an endogenous reference gene, but *GmCYP2* was more stable in these samples.

### Susceptibility level of transgenic soybean lines to *M. incognita*

The inoculum of *M. incognita* ppJ2 race 3 was obtained from the tomato cv. Santa Clara. Eggs were collected from infected roots using 100–550 μm sieves (Hussey and Barker 1973) and hatched in the dark at 28°C under aerobic conditions. Hatched ppJ2 were harvested and quantified under a microscope using counting chambers. Seeds from three transgenic soybean lines (Ev1 to Ev3) were previously germinated on Germitest paper, and uniform plants were transplanted to 280 cm^3^ conical tubes containing soil and substrate mixture (2:1; w:w). Plants were inoculated with 1,000 freshly hatched ppJ2s of *M. incognita* and maintained under greenhouse conditions at 25 ± 10°C and 50% humidity. The plants were irrigated and fertilized according to standard soybean cultivation practices as described above. Twelve plants per transgenic soybean line from the T_4_ generation were used for each treatment, and non-transgenic plants cv. BR143465 were used as a negative control. Each inoculated plant was considered a biological replicate. At 60 DAI, the plants were evaluated for the number of galls, egg masses, and eggs per gram of root, and the nematode reproduction factor was calculated. Sixty DAI represents two infectious life cycles of *M. incognita* in soybean. The reproduction factor of *M. incognita* was determined using the following Oostenbrink’s formula: nematode reproduction factor = final number of hatched ppJ2/initial number of ppJ2 or population final of nematode/initial population of nematode (Oostenbrink 1966; Windham and Williams 1987). For morphological analyses, galls from transgenic soybean plants and non-transgenic plants cv. BR143465 were dissected at 45 DAI and fixed in 2% glutaraldehyde. The fixed galls were then gradually dehydrated in 30%, 50%, 70%, 80%, and 100% ethanol and embedded in Technovit 7100 (Kulzer GmbH, Wehrheim, Germany), according to the manufacturer’s protocol. Subsequently, the galls were sectioned at 3 µm and stained in 0.05% toluidine blue. Slides were mounted with DPX mountant (Distyrene, Plasticizer, and Xylene; Sigma-Aldrich) and observed under brightfield microscopy using an AxioCamHRc digital camera (Carl Zeiss, Jena, Germany). For each transgenic line and non-transgenic plants, at least 10 galls from a minimum of five different plants were sectioned and analyzed, and the selected images are representative.

### Statistics

Data were evaluated for statistically significant differences among treatments using one-way ANOVA with Tukey’s HSD test, with a *P*-value < 0.05. All tests were performed using the SPSS Statistics software version 27 (IBM, NY, USA).

## Results

### *GmAPC7-CT* gene

The native *GmAPC7* gene (Glyma.15G096000, coding sequence of 1,683 nucleotides and 561 amino acids) has been shown to be consistently expressed in multiple soybean tissues (Almeida-Silva et al. 2023). It is an ortholog of the *AtAPC7* (At2g39090) and *AtAPC7-CT* (Araújo-Lopes et al. 2025) genes of *A. thaliana* (Fig. 1a). The coding sequence of the *GmAPC7-CT* gene corresponds to 624 nucleotides, which encode a 207-amino acid protein, with 100% identity to the C-terminal region of the GmAPC7, 81.16% identity to AtAPC7-CT, and 87.94% identity to the viral replication inhibitor (IVR) of tobacco (Suppl. File S1). Therefore, the GmAPC7-CT sequence is quite similar to the AtAPC7-CT (Araújo-Lopes et al. 2025) and NtAPC7-CT or IVR (Akad et al. 1999, 2005) proteins, in terms of conserved domains (PTHR12558:SF36, IPR011990, and GO:0005680), predicted subcellular localization (cytoplasm and nucleus), and identified as being a soluble protein (Suppl. Table S2). Furthermore, these three proteins share a conserved tertiary structure, which suggests evolutionary and functional conservation (Fig. 1b, f; Suppl. Fig. S2a and S2b).

### Successful generation of transgenic soybean lines

**Fig. 2:**
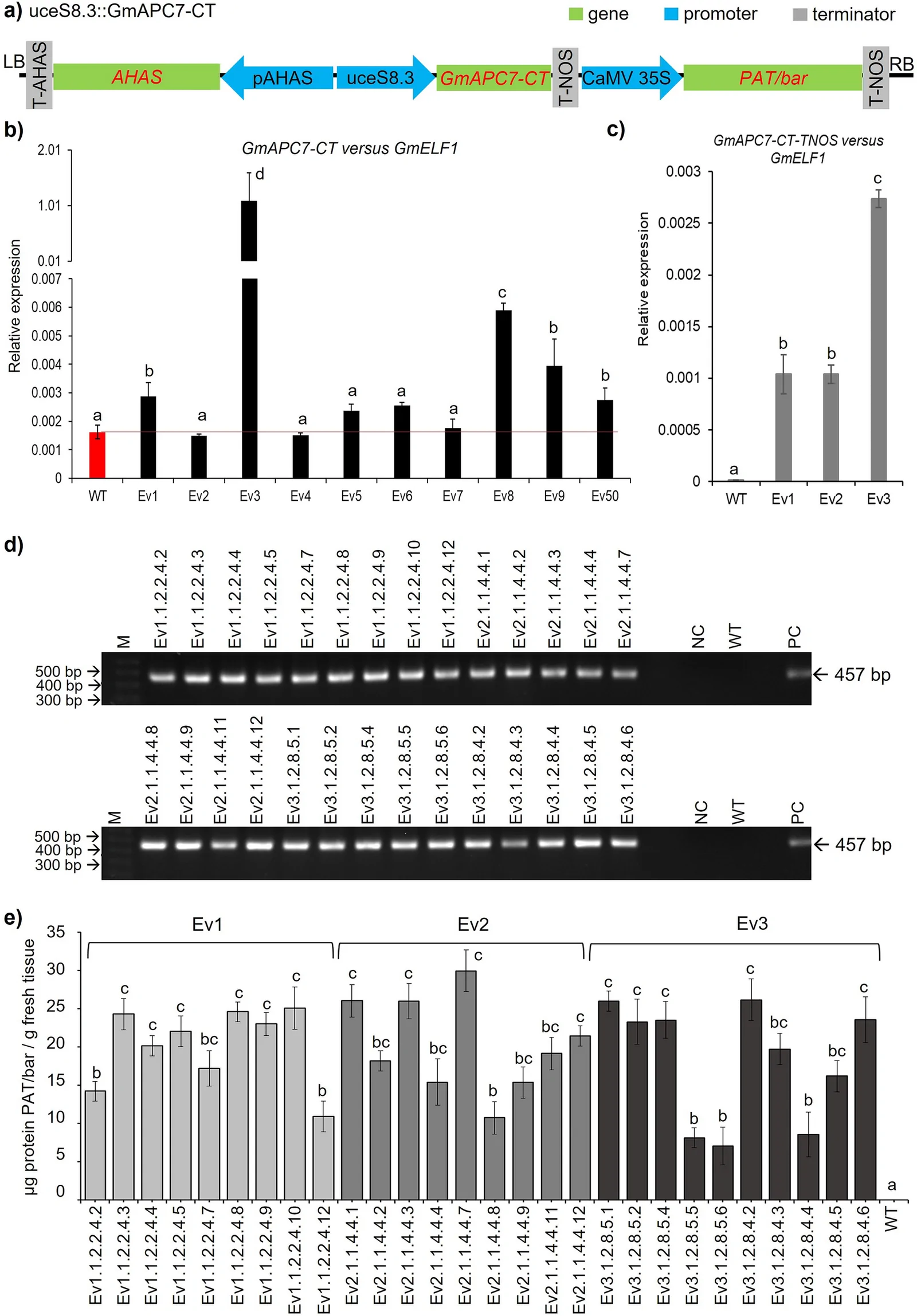
Stable transgenic lines overexpressing the *GmAPC7-CT* gene. **a** T-DNA organization cloned in a binary vector, which was used for genetic transformation of soybean embryonic axes and overexpression of the *GmAPC7-CT* coding sequence. **b**, **c** Relative expression of the *GmAPC7-CT* gene (**b**) and *GmAPC7-CT-TNOS* transcript (**c**) in transgenic soybean lines compared with non-transgenic (WT) soybean plants. Relative expression was represented by values calculated with the formula 2^−∆Ct^ using *GmELF1* as the endogenous reference gene. Error bars indicate confidence intervals from three biological replicates, each biological replicate consisting of three plants. **d** PCR detection of the transgene inserted into T_4_ transgenic soybean lines, using specific primers from the pUceS8.3::GmAPC7-CT sequence (457 bp amplicon). Weight marker: 1.0 kb DNA Ladder (Invitrogen, Cat. 10,787,018); NC: negative control; WT: non-transgenic soybean plant used as a negative control for PCR analyses and bioassays performed under greenhouse conditions; PC: aliquot of binary vector used as a positive control for PCR analysis. **e** Indirect ELISA immunodetection of PAT/bar protein in leaves of transgenic soybean lines compared to non-transgenic plants cv. BR143465. Error bars represent the standard deviation of three technical replicates per plant. Different letters on the bars indicate statistically significant differences with a *P*-value < 0.05, as determined by one-way ANOVA and Tukey’s HSD test

Ten transgenic soybean events were successfully generated with the pUceS8.3::GmAPC7-CT binary vector (Fig. 2a) from embryonic axes under in vitro selection with the Imazapyr herbicide. The T_0_ to T_4_ generations were advanced in the greenhouse at 25 ± 10°C and 50% humidity, and transgene insertion in these transgenic lines was confirmed by PCR with specific primers and by indirect ELISA using an anti-PAT/bar polyclonal antibody. The expression of the *GmAPC7-CT* gene in transgenic soybean lines was determined by real-time PCR and revealed that five of 10 lines exhibited higher expression levels compared to non-transgenic soybean plants (Fig. 2b). Furthermore, the *GmAPC7-CT-TNOS* transcript was also confirmed to be more highly expressed in the transgenic soybean lines Ev1 to Ev3 (Fig. 2c). The PCR data demonstrated the insertion and inheritance of the transgene from the T_0_ to T_4_ generations (Fig. 2d). Indirect ELISA data from the same transgenic soybean plants (Ev1 to Ev3 lines) confirmed transgene insertion and high protein expression, ranging from 7 to 30 µg of PAT/bar protein per gram of fresh leaf tissue (Fig. 2e). Therefore, several soybean transgenic lines were successfully generated, acclimated in the greenhouse, and molecularly and serologically confirmed, with the T_0_ to T_4_ generations advanced.

### Improved agronomic performance of transgenic soybean plants

**Fig. 3:**
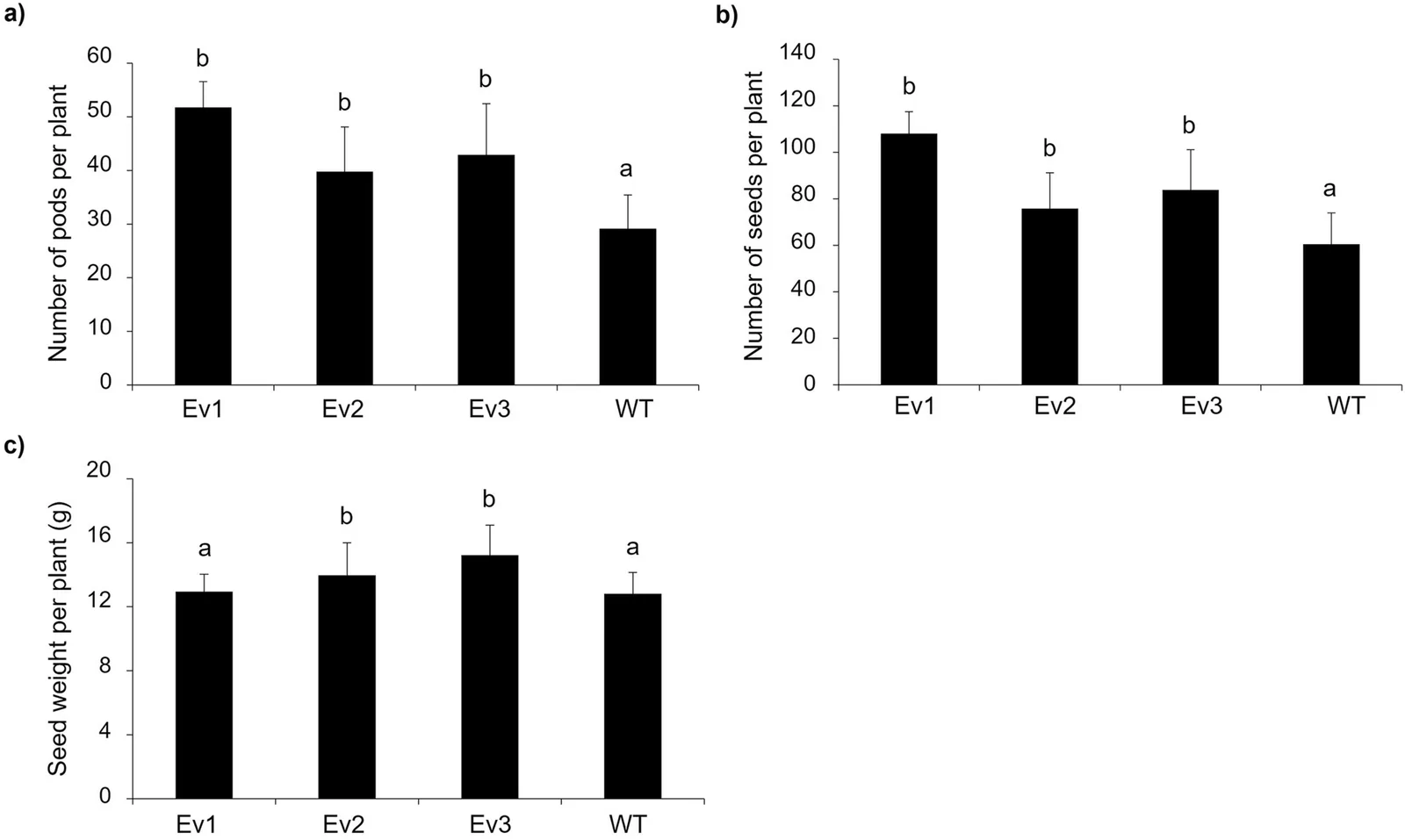
Seed yield of transgenic soybean lines maintained under greenhouse conditions. **a-c** Number of pods per plant (**a**), number of seeds per plant (**b**), and seed weight per plant (**c**) in transgenic soybean plants compared with non-transgenic (WT) plants. Error bars represent confidence intervals based on 20 biological replicates, with each biological replicate comprising one plant. Different letters on the bars indicate statistically significant differences with a *P*-value < 0.05, as determined by one-way ANOVA and Tukey’s HSD test

The plant development and seed yield of the transgenic soybean lines grown under greenhouse conditions were evaluated. There was no significant difference in the vegetative and root biomass between transgenic lines and non-transgenic soybean plants (Suppl. Fig. S3a and S3b). However, three transgenic soybean lines (Ev1 to Ev3) showed significant differences in reproductive development, with an increased number of pods per plant from 26.6 to 43.6% (Fig. 3a), number of seeds per plant from 20.2 to 44% (Fig. 3b), and seed weight per plant from 8.6 to 16% (Fig. 3c) compared with non-transgenic soybean plants. These three transgenic lines with the highest seed yield were subsequently evaluated for their susceptibility to pathogens.

### Reduced susceptibility of transgenic soybean plants to soybean mosaic virus

**Fig. 4:**
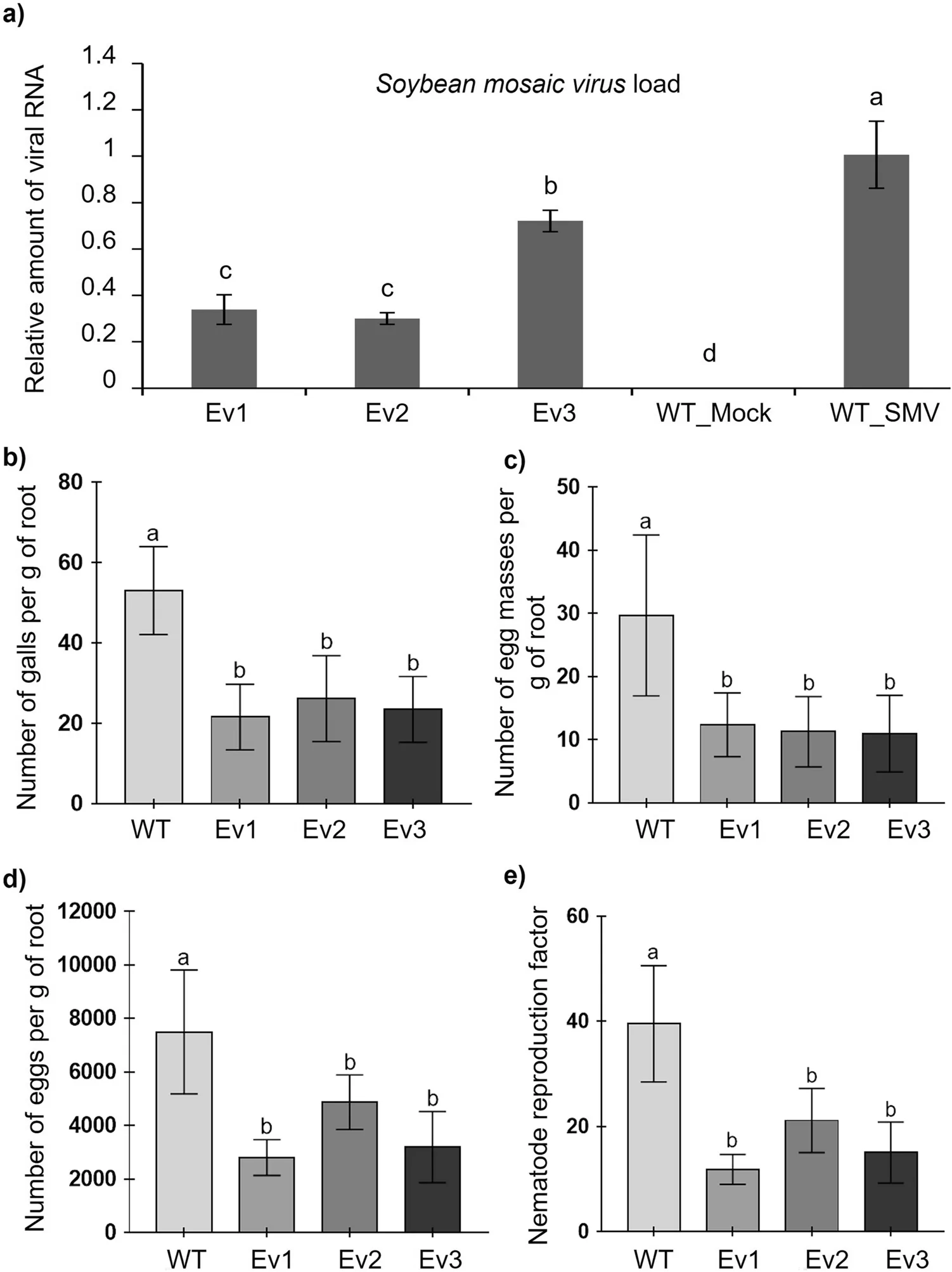
Susceptibility level of transgenic soybean lines to soybean mosaic virus and *M. incognita*. **a** Relative amount of viral RNA in transgenic soybean plants inoculated with soybean mosaic virus compared to non-transgenic (WT, cv. BR143465) plants inoculated only with buffer (mock-inoculated) or with soybean mosaic virus at 20 days after inoculation (DAI) and maintained under greenhouse conditions. The relative amount is represented by values calculated using the formula 2^−∆Ct^ with *GmCYP2* as the endogenous reference gene. Error bars represent confidence intervals based on three biological replicates, each comprising seven plants. **b** Number of nematode galls per gram of root, **c** number of nematode egg masses per gram of root, **d** number of nematode eggs per gram of root, and **e** nematode reproduction factor in transgenic soybean plants compared to non-transgenic (WT) plants cv. BR143465 at 60 DAI, maintained under greenhouse conditions. Error bars represent confidence intervals based on 12 biological replicates, with each biological replicate comprising one plant. Different letters on the bars indicate statistically significant differences with a *P*-value < 0.05, as determined by one-way ANOVA and Tukey’s HSD test

Transgenic and non-transgenic plants were successfully inoculated with soybean mosaic virus, total RNA was isolated, and cDNA was prepared. Real-time PCR data, generated in three transgenic lines (Ev1 to Ev3) mechanically inoculated with soybean mosaic virus, showed that these plants had 30–70% less viral RNA load compared with non-transgenic plants at 20 DAI (Fig. 4a). Therefore, these data revealed that the three transgenic soybean lines had reduced susceptibility to soybean mosaic virus.

### Reduced susceptibility of transgenic soybean plants to *M. incognita*

**Fig. 5:**
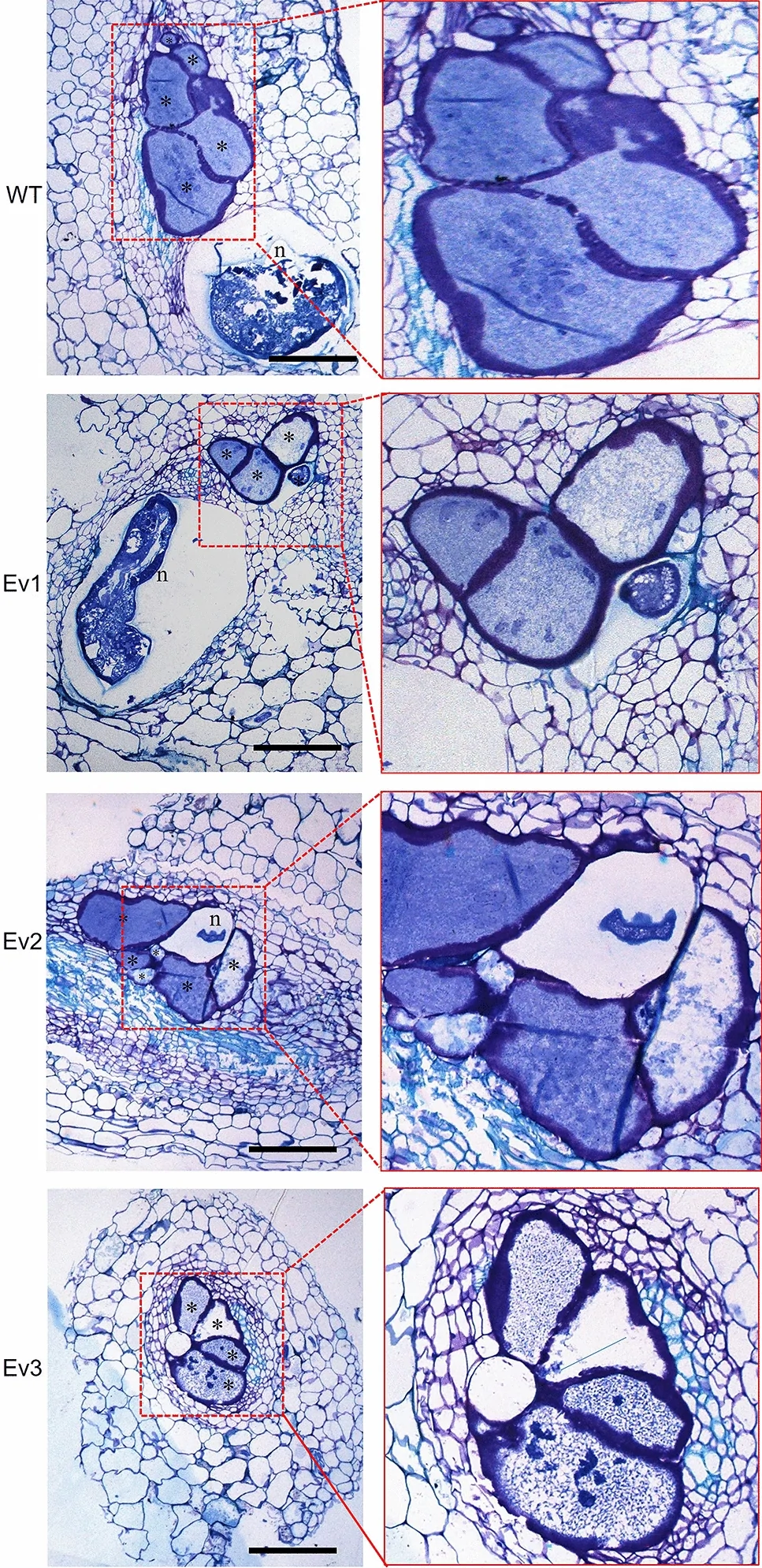
Histological morphology of galls induced by *M. incognita* in transgenic soybean lines. The left column represents unzoomed images of the roots with galls, and the right column represents zoomed-in images of the nematode feeding site morphology 45 days after inoculation (DAI). Transgenic soybean lines and non-transgenic (WT) plants were inoculated with ppJ2s of *M. incognita*, and the gall and nematode morphology were evaluated at 45 DAI. *, giant cell; n, nematode. Bars: 50 µm

Transgenic lines (Ev1 to Ev3) were challenged with *M. incognita,* which exhibited reduced susceptibility compared to non-transgenic plants. Bioassays indicated a reduction in the number of galls per gram of roots in three transgenic lines, ranging from 50.7 to 59.3% (Fig. 4b; Suppl. Fig. S4a to 4f), a decrease in the number of egg masses per gram of roots from 58.2 to 63% (Fig. 4c), a reduction in the number of eggs per gram of roots from 34.9 to 62.6% (Fig. 4d), and a lower nematode reproduction factor from 39.5 to 70% (Fig. 4e). Meanwhile, morphological analyses were performed on galls collected at 45 DAI to evaluate the effect of GmAPC7-CT overexpression in transgenic soybean roots during *M. incognita* parasitism. In contrast, non-transgenic soybean plants exhibited typical giant cells with dense cytoplasm, whereas feeding cells induced in transgenic soybean plants frequently displayed a less dense cytoplasm than those in non-transgenic plants. In addition, giant cells were smaller and exhibited a lower nuclear density in histological sections, suggesting impaired feeding cell development (Fig. 5). These features were visibly observed in galls from all three transgenic soybean lines (Ev1 to Ev3) analyzed, suggesting that overexpression of the *GmAPC7-CT* gene may play a role in the ontogenesis of galls induced by *M. incognita*. Consequently, these three transgenic soybean lines demonstrated reduced susceptibility to *M. incognita* and increased seed yield, along with reduced susceptibility to soybean mosaic virus.

## Discussion

Soybean is among the main agricultural commodities worldwide, with Brazil, the USA, and Argentina among the largest producers (Tetrault 2024). It is an important raw material for many essential commercial products, including animal feed, by-products, biodiesel, and vegetable oil (Hartman et al. 2011; Hamawaki et al. 2019). Soybean demand and production are expected to increase significantly over the coming years (Basso et al. 2024; Grossi-de-Sa and Basso 2024). Phytopathogenic nematodes, viruses, fungi, and bacteria are among the main constraints to soybean productivity worldwide (Lin et al. 2022). In particular, soybean mosaic virus and *M. incognita* are common in soybean-growing areas and can reduce crop yield and quality (Hill and Whitham 2014; Mazzetti et al. 2019). However, few soybean cultivars with any resistance to these pests are currently available to farmers (Widyasari et al. 2020; Mendes et al. 2021a, 2021b; Choi et al. 2023). Thus, the development of superior conventional, transgenic-based, or genome edited-based soybean cultivars with improved agronomic characteristics, such as increased seed yield and reduced susceptibility to pathogens, will be essential to increase agricultural production and minimize the dependence on non-selective agrochemicals (Fragoso et al. 2022). Therefore, prospecting for target genes associated with key agronomic traits and developing elite soybean cultivars can help overcome these drawbacks that limit agricultural production (Basso et al. 2022a, 2022b).

In previous studies, the full-length *AtAPC7* gene has been shown to exhibit a high level of expression in different organs of *A. thaliana*, while the *AtAPC7* 3’ portion (*AtAPC7-CT*) was shown to accumulate up to three times more transcripts than the full-length gene (Eloy et al. 2006). The protein encoded by the *AtAPC7-CT* gene demonstrated 100% amino acid sequence identity with full-length AtAPC7 and 87.94% sequence identity with the tobacco IVR (Akad et al. 1999; Eloy et al. 2006; Araújo-Lopes et al. 2025). This IVR sequence was first identified in the tobacco cv. Samsun NN, which is considered resistant to tobacco mosaic virus (Akad et al. 1999). In turn, this tobacco IVR exhibits a high amino acid sequence identity with the endogenous NtAPC7-CT protein, indicating that this IVR is NtAPC7-CT itself (Palukaitis et al. 2022; Araújo-Lopes et al. 2025). Furthermore, the greater accumulation of this IVR in tobacco showed a negative correlation with viral load, indicating a close association between IVR and plant resistance (Loebenstein and Gera 1981a, 1981b; Spiegel et al. 1989). Therefore, these data suggested that the AtAPC7-CT and NtAPC7-CT proteins may also be involved in reducing plant susceptibility to pathogens. *A. thaliana* and *N. tabacum* lines overexpressing the *AtAPC7-CT* and *NtAPC7-CT* genes, respectively, showed increased seed and biomass yield, as well as reduced susceptibility to RNA viruses and phytopathogenic fungi (Akad et al. 2005; Araújo-Lopes et al. 2025). Therefore, identifying and characterizing the potential of APC7-CT in plants of agronomic interest is important at this time, such as in soybean. In this study, the *GmAPC7-CT* gene was identified in soybean and used for transgenic overexpression in soybean lines in order to evaluate its potential to improve seed yield and reduce plant susceptibility to soybean mosaic virus and *M. incognita*. Sequence data revealed that the GmAPC7-CT protein exhibits high sequence identity, conserved domains, and a tertiary structure very similar to the NtAPC7-CT, AtAPC7-CT, and tobacco IVR proteins. These sequence data suggest that GmAPC7-CT may play a role similar to that of previously characterized APC7-CT proteins, and that overexpression of *GmAPC7-CT* gene may contribute to increased seed yield and reduced susceptibility to pathogens. To evaluate this hypothesis, ten transgenic soybean events were successfully generated, and the segregating lines were advanced from T_0_ to T_4_ generations. Preliminary screening of seed productivity in a greenhouse enabled the selection of the three best transgenic lines for subsequent analysis of their susceptibility to pathogens. These three selected lines (Ev1 to Ev3) have higher pod number, seed number, and seed weight per plant, indicating that overexpression of *GmAPC7-CT* may affect soybean yield. These data in soybean lines are similar to those obtained with *A. thaliana* lines overexpressing the *AtAPC7-CT* gene. Araújo-Lopes et al. (2025) showed that *A. thaliana* overexpressing the *AtAPC7-CT* gene presented greater development since the formation of the seed embryo, earliness in the transition from the vegetative-reproductive stages, and greater seed yield (number of seeds per plant, total weight of seed per plant, and average weight of each seed) without significant structural and morphological changes in plant tissues and organs. Furthermore, these three soybean transgenic lines also showed lower susceptibility to soybean mosaic virus and *M. incognita*. The transgenic lines showed a reduced RNA load of the soybean mosaic virus at 20 DAI, as well as a lower number of galls, number of egg masses, number of eggs, and *M. incognita* reproduction factor. Also, morphological analysis of the galls showed that the giant cells induced by *M. incognita* were apparently smaller in all three transgenic soybean lines. These data on the reduced susceptibility of soybean lines are consistent with what was observed in *A. thaliana* and *N. tabacum* lines overexpressing *AtAPC7-CT* and *NtAPC7-CT* genes, respectively. Araújo-Lopes et al. (2025) demonstrated that these *A. thaliana* lines exhibit reduced susceptibility to both begomoviruses and tobraviruses. Akad et al. (2005) showed that *N. tabacum* lines of the cultivar Samsun “nn” (a tobacco variety considered susceptible to tobacco mosaic virus) exhibit faster growth and reduced susceptibility to tobacco mosaic virus and *Botrytis cinerea*. Therefore, the combined data from these studies suggest that *APC7-CT* genes may have a conserved role across different plant species. However, the specific role and network of the APC7-CT protein in improving plant development and seed yield, as well as in reducing plant susceptibility to biotic stresses, remain to be further investigated. Recent findings have speculated that the APC7-CT protein might originate from a transcriptional unit that overlaps with the full-length *APC7* gene and is transcriptionally modulated by the SHE1 transcription factor (Yoon and Palukaitis 2021; Palukaitis et al. 2022; Yoon et al. 2023). Yoon and Palukaitis (2021) and Palukaitis et al. (2022) showed that the cucumber mosaic virus 1a protein interacts with tobacco NtAPC7-CT and NtSHE1 proteins, suggesting that the virus may directly interfere with APC7-CT protein activity and modulate plant defenses to promote plant infection. Therefore, future studies will be important to determine the subcellular localization of GmAPC7-CT in the context of plant–pathogen interactions, where dynamic changes in protein localization may play an important role during pathogenesis, as well as to identify pathogen proteins that eventually interact with it and disrupt its function or localization. Since the molecular function of APC7-CT has not yet been elucidated, greater resource use efficiency and assimilate partitioning represent plausible hypotheses for transgenic soybean lines, which require further experimental validation.

In conclusion, this study provides further information on the *GmAPC7-CT* gene and reports the successful generation of transgenic soybean lines overexpressing this gene, revealing its strong potential to increase soybean yield and reduce susceptibility to two important pathogens, soybean mosaic virus and *M. incognita*. Together, our data indicate that GmAPC7-CT plays a conserved role in soybean, analogous to that observed in previous studies with AtAPC7-CT and NtAPC7-CT in *A. thaliana* and *N. tabacum*. Thus, *GmAPC7-CT* gene emerges as a promising biotechnological target for stacking increased productivity and broad-spectrum resistance in soybean. These findings provide a solid basis for future field trials involving comparisons with commercially available resistant cultivars and for extending susceptibility assays to additional soybean pathogens of agronomic relevance.

## Supplementary Information

Below is the link to the electronic supplementary material.

Supplementary file1 (DOCX 4011 KB)

## Data Availability

All data used or generated are cited or made available with supplementary material for free access.

## Abbreviations

APC/C: Anaphase-promoting complex or cyclosome
APC: Anaphase-promoting complex
DAI: Days after inoculation
IVR: Viral replication inhibitor
PAT/bar: Phosphinothricin acetyltransferase/bialaphos
ppJ2: Pre-parasitic second-stage juveniles
TRP: Tetratricopeptide repeat
