# Supplementary material for: Transgenic overexpression of GmAPC7-CT improves seed yield and reduces susceptibility to soybean mosaic virus and Meloidogyne incognita in soybean
Source: Planta. 2026 Sep 7;264(4):118. doi: 10.1007/s00425-026-05097-6 (PMC13550036; doi:10.1007/s00425-026-05097-6)
Supplement: Supplementary file 1 [file 425_2026_5097_MOESM1_ESM.docx]

**Suppl. Table S1.** Primer sequences used in PCR and real-time RT-PCR assays for the molecular characterization of transgenic soybean lines.

| **Gene** | **Primer name** | **Primer sequence (5’-3’)** | ***Tm*** | **GC%** | **Length (bp)** | **Test** |
| --- | --- | --- | --- | --- | --- | --- |
| Glyma.15G096000  *GmAPC7-CT* | GmAPC7CT(F) | CTAGCTTTGGCTGAGCTCCAT | 60 | 52 | 145 | RT-PCR |
|  | GmAPC7CT(R) | CCTCCTGCAGCATGTTAGTGG | 61 | 57 |  |  |
| *uceS8.3::GmAPC7-CT* | GmAPC7CTNOS(F) | TGGATCCTGATGCACCTGAAG | 60 | 52 | 150 | RT-PCR |
|  | GmAPC7CTNOS(R) | AATCATCGCAAGACCGGCAA | 60 | 50 |  |  |
| Glyma.05G114900  *GmELF1* | Glyma.05G114900q(F) | GACCTTCTTCGTTTCTCGCA | 60 | 50 | 161 | RT-PCR |
|  | Glyma.05G114900q(R) | CGAACCTCTCAATCACACGC | 60 | 55 |  |  |
| *PAT/bar* | GmBARq2(F) | GACATGCCAGCAGTTTGCAC | 57 | 55 | 490 | PCR |
|  | GmBARq2(R) | CAGGGAGCACTGGTCTAGGT | 57 | 60 |  |  |
| uceS8.3::GmAPC7-CT | uceS8.3:APC7(F) | CCTGGTACCATGAAGGGAAA | 58 | 50 | 457 | PCR |
|  | GmAPC7-CT1(R) | CCTCCTGCAGCATGTTAGTGG | 58 | 57 |  |  |
| Soybean mosaic virus | SMVq1(F) | GGTTAGGAAAGTGGCCGACA | 60 | 55 | 90 | RT-PCR |
|  | SMVq1(R) | GGAGCTCAGCATTCCTGGTT | 60 | 55 |  |  |
| Glyma.12G024700 *GmCYP2* | GmCYP2(F) | CGGGACCAGTGTGCTTCTTCA | 60 | 57 | 154 | RT-PCR |
|  | GmCYP2(R) | CCCCTCCACTACAAAGGCTCG | 60 | 61 |  |  |

**Suppl. Table S2.** Predicted features of the GmAPC7-CT *versus* AtAPC7-CT and NtAPC7-CT proteins.

| **AtAPC7-CT** | **NtAPC7-CT** | **GmAPC7-CT** |
| --- | --- | --- |
| PTHR12558:SF36 | PTHR12558:SF36 | PTHR12558:SF36 |
| IPR011990 | IPR011990 | IPR011990 |
| GO:0005680 | GO:0005680 | GO:0005680 |
| CDS 624 bp  Protein 207 aa | CDS 600 bp  Protein 199 aa | CDS 624 bp  Protein 207 aa |
| Cytoplasm and nucleus | Cytoplasm and nucleus | Cytoplasm and nucleus |
| Nuclear localization signal and nuclear export signal (probability: 0.6240) | Nuclear localization signal and nuclear export signal (probability: 0.5379) | Nuclear localization signal and nuclear export signal (probability: 0.5882) |
| No transmembrane domain | No transmembrane domain | No transmembrane domain |
| Membrane association: soluble (probability: 0.8500) | Membrane association: soluble (probability: 0.8480) | Membrane association: soluble (probability: 0.8500) |

**Suppl. Fig. S1** Phenotype of soybean plants of genotype PI595099 (symptomatic susceptible) and cultivar BRS133 (asymptomatic susceptible) used as controls for inoculation with soybean mosaic virus (SMV) at 12 to 18 days after inoculation (DAI). Red arrow. one of the inoculated leaves.

**Suppl. Fig. S2** Comparison of the tertiary structure of the (**a**) GmAPC7-CT *versus* AtAPC7-CT and (**b**) GmAPC7-CT *versus* NtAPC7-CT or tobacco IVR proteins provided by AlphaFold v.3.

**Suppl. Fig. S3** Vegetative and root biomass of transgenic soybean lines maintained under greenhouse conditions. **a** Vegetative biomass per plant and (**b**) root biomass per plant in transgenic soybean plants compared with non-transgenic (WT) plants. Error bars represent confidence intervals based on 20 biological replicates, with each biological replicate comprising one plant. Different letters on the bars indicate statistically significant differences with a *P*-value <0.05, as determined by one-way ANOVA and Tukey’s HSD test.

**Suppl. Fig. S4** Phenotype of galls induced by *M. incognita* in roots of transgenic soybean lines and non-transgenic (WT) plants at 60 days after inoculation. The images show (**a**, **b**) multiple well-developed root swellings in WT plants cv. BR143465 and WT plants cv. BRS133 (nematode-susceptible cultivar) compared with the reduced number and smaller size of root knots observed in (**c**) WT plants of the genotype PI595099 (nematode-resistant cultivar) and (**d**, **f**) soybean transgenic lines (Ev1 to Ev3).

**Suppl. File S1.** Full-length *APC7* gene and protein sequences of soybean, *A. thaliana*, and tobacco, highlighting the position of the *GmAPC7-CT* gene.

**>*GmAPC7* (*Glyma.15G096000*) gene and *GmAPC7-CT* gene (yellow)**
ATGGAAGTTCCGAAGGATCAAATCGCCACTCTTCTCGAACATGGCCTATACAACTCCGCTCAGATGCTTGGTTGTTTTCTTGTTTCTTCACCTGCTGTTAATGCTGAATCTGCCGCACACCTCAAAACTGAGAGCTTGGTGCTACTTGGTGATTCTTTCTTCCGTGAAAGGGAGTATCGTAGAGCCATTCACACCTACAAACAAGCTTTGCAATGCTACAAGATGATCCCAAAACAGAACATGACATCTTCTCGGAGTTCATTGTCATCAAACCGGTCTTCGTCACCGAATTCCTGCAATGGATCAGTGATCAATGAGAATGAGGTGAAGTTCAAAATTGCATCATGTCACTGTTTTCTGAACGAGAATAAAGCAGCTCTAGTTGAGATGGAAGGCATTCCAAGTAAAGCTAGAAATTTACCGATGAATTTGTTATTAGGGAAGCTATATCGAATTTCTAGACATAGCCGAGCTGCTGTTGCTATTTACAAAGAATGTCTAAGACATTGTCCTTTTGTACTTGAGGCTATCACAGCTTTAGCAGAACTTGGATCCACTGCAAAGGACATCATTACATTGATTCCTCAGACCCCGAATAGAAGTGGTAGAGCTTCATTTGATCATACTGATTCAAGTCGTTGGCTTCAACGCTATGTTGAAGCTCAATGTTGCATGGCTTCAAATGATTACAAAGGTGGATTGGAACTCTTTGCAGATCTTTTACAGCGTTTCCCTAATAATATACACTTACTACTTGAGATGGCAAAGGTTGAAGCTATTATTGGAAAGAATGAAGAGGCCATTATGAATTTTGAAAAGGCCCGTTCAATTGATCCATACATCATAACATATATGGATGAGTATGCAATGCTTCTAAAGCTAAAGTCTGATTATCCAAAGCTGAACAAGTTAGTGCATGATTTATTGAATATTGATCCTGCAAGGCCTGAGGTTTTTGTAGCTTTGTCTGTTTTGTGGGAAAGGAAAGATGAGAAAAAAGCATTACAATATGCTGAGCAGAGTATTCGGATCGATGAGAGGCACATACCAGGGTATATAATGAAGGGAAATTTATTATTGACGATGAAACGGGCAGAAGCAGCTGTGCCTGCCTTCAGAGCAGCTCAAGAATTGAGACCTGATATTCGCTCATATCAAGGTTTGGTCCATACATATTTGGCTCTCTCTAAAATCAAAGAGGCTCTGTATGCTTCAAGAGAAGCTATGAAAGCAATGCCTCAGTCGGCAAAGGCTTTAAAATTAGTAGGTGATGTGCATGCCAGTAATTCTAGTGGCAGAGAAAAGGCTAAAAAATTCTACGAGTCTGCACTAAGACTGGAACCTGGTTACCTTGGAGCTGCACTAGCTTTGGCTGAGCTCCATGTTATTGAGGGCCGAAATGGAGATGCTGTGTCTCTGCTTGAGCGATATCTTAAAGACTGGGCTGATGATTCGCTTCATGTAAAACTTGCACAAGTTTTTGCAGCCACTAACATGCTGCAGGAGGCTTTGTCACATTATCAGGCTGCATTAAGGTTAAACCCCCAGAATGAAGCAGCAAAACGGGGTTTAGAGCGATTAGAGAAGCAAATGAAGGGAGTGGATCCTGATGCACCTGAAGAAGATGAAGACAATGATGTTGAAGATGCAGACGGAGATCAAGACGAGACTGAACTTCTGTGA

**>*GmAPC7* (*Glyma.15G096000*) gene and *GmAPC7-CT* gene (yellow)**
atggaagttccgaaggatcaaatcgccactcttctcgaacatggcctatacaactccgct

```
 M  E  V  P  K  D  Q  I  A  T  L  L  E  H  G  L  Y  N  S  A
cagatgcttggttgttttcttgtttcttcacctgctgttaatgctgaatctgccgcacac
 Q  M  L  G  C  F  L  V  S  S  P  A  V  N  A  E  S  A  A  H
ctcaaaactgagagcttggtgctacttggtgattctttcttccgtgaaagggagtatcgt
 L  K  T  E  S  L  V  L  L  G  D  S  F  F  R  E  R  E  Y  R
agagccattcacacctacaaacaagctttgcaatgctacaagatgatcccaaaacagaac
 R  A  I  H  T  Y  K  Q  A  L  Q  C  Y  K  M  I  P  K  Q  N
atgacatcttctcggagttcattgtcatcaaaccggtcttcgtcaccgaattcctgcaat
 M  T  S  S  R  S  S  L  S  S  N  R  S  S  S  P  N  S  C  N
ggatcagtgatcaatgagaatgaggtgaagttcaaaattgcatcatgtcactgttttctg
 G  S  V  I  N  E  N  E  V  K  F  K  I  A  S  C  H  C  F  L
aacgagaataaagcagctctagttgagatggaaggcattccaagtaaagctagaaattta
 N  E  N  K  A  A  L  V  E  M  E  G  I  P  S  K  A  R  N  L
ccgatgaatttgttattagggaagctatatcgaatttctagacatagccgagctgctgtt
 P  M  N  L  L  L  G  K  L  Y  R  I  S  R  H  S  R  A  A  V
gctatttacaaagaatgtctaagacattgtccttttgtacttgaggctatcacagcttta
 A  I  Y  K  E  C  L  R  H  C  P  F  V  L  E  A  I  T  A  L
gcagaacttggatccactgcaaaggacatcattacattgattcctcagaccccgaataga
 A  E  L  G  S  T  A  K  D  I  I  T  L  I  P  Q  T  P  N  R
agtggtagagcttcatttgatcatactgattcaagtcgttggcttcaacgctatgttgaa
 S  G  R  A  S  F  D  H  T  D  S  S  R  W  L  Q  R  Y  V  E
gctcaatgttgcatggcttcaaatgattacaaaggtggattggaactctttgcagatctt
 A  Q  C  C  M  A  S  N  D  Y  K  G  G  L  E  L  F  A  D  L
ttacagcgtttccctaataatatacacttactacttgagatggcaaaggttgaagctatt
 L  Q  R  F  P  N  N  I  H  L  L  L  E  M  A  K  V  E  A  I
attggaaagaatgaagaggccattatgaattttgaaaaggcccgttcaattgatccatac
 I  G  K  N  E  E  A  I  M  N  F  E  K  A  R  S  I  D  P  Y
atcataacatatatggatgagtatgcaatgcttctaaagctaaagtctgattatccaaag
 I  I  T  Y  M  D  E  Y  A  M  L  L  K  L  K  S  D  Y  P  K
ctgaacaagttagtgcatgatttattgaatattgatcctgcaaggcctgaggtttttgta
 L  N  K  L  V  H  D  L  L  N  I  D  P  A  R  P  E  V  F  V
gctttgtctgttttgtgggaaaggaaagatgagaaaaaagcattacaatatgctgagcag
 A  L  S  V  L  W  E  R  K  D  E  K  K  A  L  Q  Y  A  E  Q
agtattcggatcgatgagaggcacataccagggtatataatgaagggaaatttattattg
 S  I  R  I  D  E  R  H  I  P  G  Y  I  M  K  G  N  L  L  L
acgatgaaacgggcagaagcagctgtgcctgccttcagagcagctcaagaattgagacct
 T  M  K  R  A  E  A  A  V  P  A  F  R  A  A  Q  E  L  R  P
gatattcgctcatatcaaggtttggtccatacatatttggctctctctaaaatcaaagag
 D  I  R  S  Y  Q  G  L  V  H  T  Y  L  A  L  S  K  I  K  E
gctctgtatgcttcaagagaagctatgaaagcaatgcctcagtcggcaaaggctttaaaa
 A  L  Y  A  S  R  E  A  M  K  A  M  P  Q  S  A  K  A  L  K
ttagtaggtgatgtgcatgccagtaattctagtggcagagaaaaggctaaaaaattctac
 L  V  G  D  V  H  A  S  N  S  S  G  R  E  K  A  K  K  F  Y
gagtctgcactaagactggaacctggttaccttggagctgcactagctttggctgagctc
 E  S  A  L  R  L  E  P  G  Y  L  G  A  A  L  A  L  A  E  L
catgttattgagggccgaaatggagatgctgtgtctctgcttgagcgatatcttaaagac
 H  V  I  E  G  R  N  G  D  A  V  S  L  L  E  R  Y  L  K  D
tgggctgatgattcgcttcatgtaaaacttgcacaagtttttgcagccactaacatgctg
 W  A  D  D  S  L  H  V  K  L  A  Q  V  F  A  A  T  N  M  L
caggaggctttgtcacattatcaggctgcattaaggttaaacccccagaatgaagcagca
 Q  E  A  L  S  H  Y  Q  A  A  L  R  L  N  P  Q  N  E  A  A
aaacggggtttagagcgattagagaagcaaatgaagggagtggatcctgatgcacctgaa
 K  R  G  L  E  R  L  E  K  Q  M  K  G  V  D  P  D  A  P  E
gaagatgaagacaatgatgttgaagatgcagacggagatcaagacgagactgaacttctg
 E  D  E  D  N  D  V  E  D  A  D  G  D  Q  D  E  T  E  L  L
tga
 -
```

**>GmAPC7 protein and GmAPC7-CT protein (bold)**

MEVPKDQIATLLEHGLYNSAQMLGCFLVSSPAVNAESAAHLKTESLVLLGDSFFREREYRRAIHTYKQALQCYKMIPKQNMTSSRSSLSSNRSSSPNSCNGSVINENEVKFKIASCHCFLNENKAALVEMEGIPSKARNLPMNLLLGKLYRISRHSRAAVAIYKECLRHCPFVLEAITALAELGSTAKDIITLIPQTPNRSGRASFDHTDSSRWLQRYVEAQCCMASNDYKGGLELFADLLQRFPNNIHLLLEMAKVEAIIGKNEEAIMNFEKARSIDPYIITYMDEYAMLLKLKSDYPKLNKLVHDLLNIDPARPEVFVALSVLWERKDEKKALQYAEQSIRIDERHIPGYI**MKGNLLLTMKRAEAAVPAFRAAQELRPDIRSYQGLVHTYLALSKIKEALYASREAMKAMPQSAKALKLVGDVHASNSSGREKAKKFYESALRLEPGYLGAALALAELHVIEGRNGDAVSLLERYLKDWADDSLHVKLAQVFAATNMLQEALSHYQAALRLNPQNEAAKRGLERLEKQMKGVDPDAPEEDEDNDVEDADGDQDETELL**

**>AtAPC7 protein and AtAPC7-CT protein (bold)**

MEVPKEQIATLIEHGLYDSAEMLGCFLVSSPTVSAETSPQLKAENLILLGDALFHQREHRRAIHTYKQALHHYTRIPKQSSGISRSSLSLSTRSSVNASSISAINENEVRFKIASSHFALNETKAAIAEMESVKTRSLEMNILMAKLHRNSGYNRGAIAFYKECLRQCPYVLEAVIGLAELGVSAKDIISSFTQTSNRSAKVSLDQIDPTRWLQRYVEAQCCVASHAYKGALELFAELLQRFPNNVHLLTETAKVEAIIGKNDEAIMRFEKVRSIDPYTLTSMDEYAMLLQIKCDYSRLNKLVHDLLSVDHTRAEVFVALSVLWERKDARTALSYAEKSIRVDERHIPGYI**MKGNLLLQAKRPEAAAIAFRAAQNLRSDLRSYQGLVHSYLAFGKTKEALYTAREAMNAMPQSAKALKLVGDVHAFTSSGREKAKKFYESGLRLEPGYLGAVLALAELHLMEGRNGDAVSLLERYLKDYADDSLHVKLAQVFAATNMLQDSLSHFQAALRINPQNEAAKKGLDRLEKQMKGIDPDATDENDENDVEDVDGDTEEAELM**

**>NtAPC7 protein and IVR protein sequence (bold)**

MDVPKDQMSTLLDHGLFPSAQMLGCFLVSSSSVSIETSPHLKAENLVLLGDALFREKEYKRAIHAYKQALHCHRIIPKQNATPIRSALSASTRSSSPNSFNISAINENEVKFKIASSHCALNENRAALLEMEGIPSKARNLQMNLMMGKLYRSSRHTRAAVTCYKECLRHCPYIIEAIIALAELGVAAKDIISLFPQTPTRGARSTFDHFDSSRWLQRYVEAQCCIASNDYKGGLEIFAELLRRFPCNIHILLEMAKVEAIIGKTEEAITDFEKGKTSVRSTDPYVITYMDEYAMLLKQKSDYSKLNKLVHDLLNIDPTRPEVFVALSVLWERKDERGALSYAEKSIRIDERHIPGYIMKGNLFFS**MNRPEAAVVAFRGAQELRPDLRSYQGLVRSYLAISKIKEALHAAREAMKAMPQSAKALKLVGDVHASNTSGREKAKKFYESSLRLEPGYLGAALALAELHVIEGRTVDAVTLLERYLKDWADDSLHVKLAQVFAATNMPQDALSHYQAALRINPQNEAAKKGLERLEKQLKGVDPDAPEEDEENDVEDADADQEEAELL**

**>AtAPC7, GmAPC7, and NtAPC7 proteins highlighting the IVR protein sequence (yellow)**

```
AtAPC7          MEVPKEQIATLIEHGLYDSAEMLGCFLVSSPTVSAETSPQLKAENLILLGDALFHQREHR
GmAPC7          MEVPKDQIATLLEHGLYNSAQMLGCFLVSSPAVNAESAAHLKTESLVLLGDSFFREREYR
NtAPC7          MDVPKDQMSTLLDHGLFPSAQMLGCFLVSSSSVSIETSPHLKAENLVLLGDALFREKEYK
                *:***:*::**::***: **:*********.:*. *::.:**:*.*:****::*.:.*:.

AtAPC7          RAIHTYKQALHHYTRIPKQSSGISRSSLSLSTRSSV-NASSISAINENEVRFKIASSHFA
GmAPC7          RAIHTYKQALQCYKMIPKQNMTSSRSSLS-SNRSSSPNSCNGSVINENEVKFKIASCHCF
NtAPC7          RAIHAYKQALHCHRIIPKQNATPIRSALSASTRSSSPNSFNISAINENEVKFKIASSHCA
                ****:*****: :  ****.    **:** *.***  *: . *.******.*****.*

AtAPC7          LNETKAAIAEMESV--KTRSLEMNILMAKLHRNSGYNRGAIAFYKECLRQCPYVLEAVIG
GmAPC7          LNENKAALVEMEGIPSKARNLPMNLLLGKLYRISRHSRAAVAIYKECLRHCPFVLEAITA
NtAPC7          LNENRAALLEMEGIPSKARNLQMNLMMGKLYRSSRHTRAAVTCYKECLRHCPYIIEAIIA
                ***..**: ***.:  *:*.* **:::.**:* * :.*.*:: ******:**:::**: .

AtAPC7          LAELGVSAKDIISSFTQTSNRSAKVSLDQIDPTRWLQRYVEAQCCVASHAYKGALELFAE
GmAPC7          LAELGSTAKDIITLIPQTPNRSGRASFDHTDSSRWLQRYVEAQCCMASNDYKGGLELFAD
NtAPC7          LAELGVAAKDIISLFPQTPTRGARSTFDHFDSSRWLQRYVEAQCCIASNDYKGGLEIFAE
                ***** :*****: :.**..*... ::*: *.:************:**: ***.**:**:

AtAPC7          LLQRFPNNVHLLTETAKVEAIIGKNDEAIMRFEK----VRSIDPYTLTSMDEYAMLLQIK
GmAPC7          LLQRFPNNIHLLLEMAKVEAIIGKNEEAIMNFEK----ARSIDPYIITYMDEYAMLLKLK
NtAPC7          LLRRFPCNIHILLEMAKVEAIIGKTEEAITDFEKGKTSVRSTDPYVITYMDEYAMLLKQK
                **.*** *:*:* * *********.:***  ***    .** *** :* ********: *

AtAPC7          CDYSRLNKLVHDLLSVDHTRAEVFVALSVLWERKDARTALSYAEKSIRVDERHIPGYIMK
GmAPC7          SDYPKLNKLVHDLLNIDPARPEVFVALSVLWERKDEKKALQYAEQSIRIDERHIPGYIMK
NtAPC7          SDYSKLNKLVHDLLNIDPTRPEVFVALSVLWERKDERGALSYAEKSIRIDERHIPGYIMK
                .**..*********.:* :*.************** . **.***:***:***********

AtAPC7          GNLLLQAKRPEAAAIAFRAAQNLRSDLRSYQGLVHSYLAFGKTKEALYTAREAMNAMPQS
GmAPC7          GNLLLTMKRAEAAVPAFRAAQELRPDIRSYQGLVHTYLALSKIKEALYASREAMKAMPQS
NtAPC7          GNLFFSMNRPEAAVVAFRGAQELRPDLRSYQGLVRSYLAISKIKEALHAAREAMKAMPQS
                ***::  :*.***. ***.**:**.*:*******.:***:.* ****:::****:*****

AtAPC7          AKALKLVGDVHAFTSSGREKAKKFYESGLRLEPGYLGAVLALAELHLMEGRNGDAVSLLE
GmACP7          AKALKLVGDVHASNSSGREKAKKFYESALRLEPGYLGAALALAELHVIEGRNGDAVSLLE
NtAPC7          AKALKLVGDVHASNTSGREKAKKFYESSLRLEPGYLGAALALAELHVIEGRTVDAVTLLE
                ************ .:************.**********.*******::***. ***:***

AtAPC7          RYLKDYADDSLHVKLAQVFAATNMLQDSLSHFQAALRINPQNEAAKKGLDRLEKQMKGID
GmAPC7          RYLKDWADDSLHVKLAQVFAATNMLQEALSHYQAALRLNPQNEAAKRGLERLEKQMKGVD
NtAPC7          RYLKDWADDSLHVKLAQVFAATNMPQDALSHYQAALRINPQNEAAKKGLERLEKQLKGVD
                *****:****************** *::***:*****:********.**:*****:**:*

AtAPC7          PDATDENDENDVEDVDGDTEEAELM
GmAPC7          PDAPEEDEDNDVEDADGDQDETELL
NtAPC7          PDAPEEDEENDVEDADADQEEAELL
                ***.:*:::*****.*.* :*:**:
```
